# Comparison between leached metakaolin and leached diatomaceous earth as raw materials for the synthesis of ZSM-5

**DOI:** 10.1186/2193-1801-3-292

**Published:** 2014-06-10

**Authors:** Wilson Aguilar-Mamani, Gustavo García, Jonas Hedlund, Johanne Mouzon

**Affiliations:** Chemical Technology, Luleå University of Technology, Luleå, Sweden; Department of Chemistry, Faculty of Science and Technology, San Simon University, Cochabamba, Bolivia; Solid State and Theoretical Chemistry, Chemistry Research Institute, San Andres Mayor University, University Campus, Cota Cota, 27 Street, La Paz, Bolivia

**Keywords:** Kaolin, Diatomaceous earth, n-butylamine, ZSM-5 zeolite

## Abstract

Inexpensive raw materials have been used to prepare ZSM-5 zeolites with SiO_2_/Al_2_O_3_ molar ratios in the range 20 – 40. Kaolin or Bolivian diatomaceous earth was used as aluminosilicate raw materials and sodium hydroxide and *n-*butylamine were used as mineralizing agents and template. Dealumination of the raw materials by acid leaching made it possible to reach appropriate SiO_2_/Al_2_O_3_ ratios and to reduce the amount of iron and other impurities. After mixing the components and aging, hydrothermal treatment was carried out and the products were recovered The results clearly show for the first time that well-crystallized ZSM-5 can be directly prepared from leached metakaolin or leached diatomaceous earth using sodium hydroxide and *n*-butylamine as mineralizing agents and template under appropriate synthesis conditions. A longer induction time prior to crystallization was observed for reaction mixtures prepared from leached diatomaceous earth, probably due to slower digestion of the fossilized diatom skeletons as compared with that for microporous leached metakaolin. The use of leached diatomaceous earth allowed higher yield of ZSM-5 crystals within comparable synthesis times. However, low amounts of Mordenite formed, which was related to the high calcium content of diatomaceous earth. Another considerable advantage of diatomaceous earth over kaolin is that diatomaceous earth does not require heat treatment at high temperature for metakaolinization.

## Introduction

The zeolite ZSM-5 (Zeolites Socony Mobil) is an aluminosilicate with high silica ratio with suitable properties for catalysis, adsorption and membrane applications (Jacobs 1981, Tavolaro and Drioli 1999, Weitkamp 2000). Researchers (Argauer and Landolt 1972) of Mobil Oil Corporation obtained the first patent on the synthesis of ZSM-5 zeolite, in which they described that this zeolite can be formed with molar ratios of SiO_2_/Al_2_O_3_ varying between 20 and 120. Depending on this ratio, the acidity and surface properties of ZSM-5 vary and therefore it is important to carefully control this parameter in the final product (Armaroli et al. 2006, Shirazi et al. 2008).

Typical syntheses of ZSM-5 require sources of silicon and aluminium, a mineralizer (e.g. OH^−^ or F^−^) and an organic molecule as templating agent. Quaternary ammonium compounds like tetrapropyl ammonium bromide (TPA-Br) (Padovan et al. 1984, Shirazi et al. 2008) and tetrapropyl ammonium hydroxide (TPA-OH) (Kotasthane and Shiralkar 1986) are mostly used for the synthesis of ZSM-5. Unfortunately, these quaternary ammonium compounds are rather expensive. The molecule *n*-butylamine was reported (Sang et al. 2004, Zhao 2005, Martins et al. 2006, Feng et al. 2009) as an alternative templating agent to replace TPA-Br and TPA-OH and is about 100 times less expensive on a molar basis. In the past two decades, efforts have also been undertaken to identify inexpensive Si and Al sources to synthesize ZSM-5(Chareonpanich et al. 2004, Sanhueza et al. 2004, Mignoni et al. 2007, Aliev et al. 2011) and it has been shown that kaolin clay and diatomaceous earth are two suitable and inexpensive sources of silica and alumina.

Kaolin clay contains kaolinite with a SiO_2_/Al_2_O_3_ molar ratio close to 2 and therefore it is well suited for the preparation of low-silica zeolites such as zeolite A (Costa et al. 1988, Murat et al. 1992, Chandrasekhar et al. 1997, Sanhueza et al. 1999). To obtain this zeolite from kaolin, two steps are necessary: first, a thermal treatment of kaolin to obtain an amorphous and reactive material denoted metakaolin. The second step is a hydrothermal treatment to convert metakaolin to zeolite in an alkaline aqueous medium. Preparation of zeolites with higher SiO_2_/Al_2_O_3_ molar ratios such as zeolites X (De Lucas et al. 1992, Caballero et al. 2007, Colina and Llorens 2007) and Y (Bosch et al. 1983, Atta et al. 2007) from kaolinite has also been reported. However, the syntheses of these zeolites require either an increase of the amount of silica or partial removal of aluminium. The first alternative implies using an additional source of silica with high solubility, e.g. sodium silicate. The second alternative, i.e. dealumination, consists in either leaching kaolin in a solution of an inorganic acid (HCl, H_2_SO_4_, HNO_3_) (Ford 1992) or alternatively calcining the kaolin with an inorganic acid (H_2_SO_4_) (Colina et al. 2001, Colina et al. 2002)

The synthesis of ZSM-5 zeolite from kaolin with additional sources of silica has been reported in patent (Reid 1982) and research papers (Khatamian and Irani 2009, Kovo et al. 2009). Dealumination of metakaolinite to synthesize ZSM-5 has also been investigated (Madhusoodana et al. 2005); (Zhang et al. 2007); (Madhusoodana et al. 2001). In all these studies, expensive tetrapropylamine was used as template. The synthesis of ZSM-5 with a high SiO_2_/Al_2_O_3_ molar ratio from metakaolin and silica sol and less expensive *n*-butylamine has been reported (Feng et al. 2009). However, to the best of our knowledge, a combination of dealumination of metakaolin by acid treatment together with the use of *n-*butylamine as a template has not yet been reported.

Diatomaceous earth is another inexpensive source of silica, which is a sedimentary rock comprised of fossilized skeletal remains of diatoms. It consists essentially of amorphous hydrated silica and a small amount of alumina and also impurities such as iron (Sanhueza et al. 2003). It can be used to produce mesoporous material such as MCM-41 (Sanhueza et al. 2006) and also both low and high silica zeolites such as A (Ghosh et al. 1994), P (Wajima et al. 2008) or NaP (Wajima et al. 2006), analcime (Chaisena and Rangsriwatananon 2005); (Rangsriwatananon et al. 2008), cancrinite (Chaisena and Rangsriwatananon 2005), hydroxisodalite (Chaisena and Rangsriwatananon 2005), NaY (Chi et al. 2011) and mordenite (Sanhueza et al. 2003). In most of these syntheses, the raw diatomaceous earth was acid treated to remove iron and other impurities. The conversion of diatomaceous earth to ZSM-5 was also studied in combination with other raw materials such as paper sludge ash (Wajima et al. 2008) or volcanic ash (Aliev et al. 2011). However, there are a few studies on the synthesis of ZSM-5 by using only diatomaceous earth as silica source. In these studies, diethanolamine(Sanhueza et al. 2004) and expensive tetrapropylammonium bromide (Shan et al. 2004) were used as templates and these synthesis required quite long crystallization times from 40 hours to 6 days at a quite high temperature of 180°C.

In the present work, we show for the first time that leached metakaolinite or diatomaceous earth in combination with sodium hydroxide and *n-*butylamine can be used as inexpensive raw materials for the synthesis of ZSM-5 without using an additional source of silica. However, both sources of alumino-silica are shown to behave differently during the course of synthesis and to lead to slightly different reaction products. In particular, we discuss these discrepancies in terms of composition, morphology, and porosity of the raw materials.

## Experimental

### Raw materials

Kaolin (Riedel de Haen, pro analysi), diatomaceous earth (Murmuntani zone in the locality of Llica, Potosi, Bolivia), sodium hydroxide (Sigma Aldrich, reagent grade, ≥98%, anhydrous pellets), *n-*butylamine (Sigma Aldrich, 99.5%) and hydrochloric acid (Merk, pro analysi 37%) were used as reagents.

### Heat treatment

Kaolin was first calcined in a porcelain crucible that was placed in a furnace and heated at a rate of 8°C/min in air. When the temperature reached 750°C, this temperature was maintained for 2 h to obtain metakaolin and the temperature in the furnace was then reduced to room temperature. It was not necessary to carry out the heat treatment for the diatomaceous earth in order to obtain ZSM-5, and consequently, this material was not heat treated. On the other hand, if the heat treatment of kaolin was omitted, no zeolite formed.

### Dealumination of raw materials

Metakaolin and diatomaceous earth were acid leached in a spherical glass container under reflux conditions in a thermostated oil bath maintained at 115°C. Metakaolin or diatomaceous earth was stirred in hydrochloric acid (3 M) for 150 minutes. The metakaolin or diatomaceous earth to acid weight ratio was 1:17. Subsequently, the suspension was quenched and the acid leached product was washed with distilled water. Finally, the product was separated by filtration and the filter cake was washed with distilled water until the pH reached a value close to 7.

### Hydrothermal synthesis

The synthesis mixtures were prepared by mixing the aluminosilicate sources with distilled water, *n-*butylamine (NBA) and sodium hydroxide. The molar ratios in the synthesis mixtures were: Na_2_O/SiO_2_ = 0.18; SiO_2_/Al_2_O_3_ = X; SiO_2_/NBA = 7; H_2_O/SiO_2_ = 30, where X = 33 and 44 for leached metakaolin and leached diatomaceous earth, respectively. The mixtures were aged under stirring for 24 hours at room temperature and were thereafter hydrothermally heated in Teflon lined stainless steel autoclaves kept for different times in an oil bath at 165°C. After hydrothermal treatment, the solids were recovered by filtration and washed with distilled water until the pH reached a value close to 7. The powders were dried at 100°C overnight and finally calcined at 550°C for 6 hours to remove the template.

### Characterization of the products

The chemical compositions of kaolin, diatomaceous earth, leached aluminosilicates and the final products were determined using inductively coupled plasma-sector field mass spectrometry (ICP-SFMS). Samples of 0.1 g were fused with 0.4 g of LiBO_2_ and dissolved in HNO_3_. Crystallinity was examined by X-ray diffractometry (XRD) using a PANalytical Empyrean X-ray Diffractometer equipped with Cu LFF HR X-ray tube, a graphite monochromator, and a PIXcel3D detector. The X-ray tube was operated at 30 mA and 40 kV. The investigated 2θ range was from 5 to 50° with a step size of 0.026°. The degree of crystallinity was calculated by using the area of characteristic peaks of ZSM-5 between 22 and 25° after background removal following the equation by van Hooff (Vanhooff et al. 1991).

ZSM-5 crystals with an average length of 10 μm synthesized from silicic acid and TPAOH by following the method reported by Lechert and Kleinwort (Robson and Lillerud 2001) were used as standard.

The morphology of the ZSM-5 crystals was studied by scanning electron microscopy (SEM, Magellan 400, FEI Company) without coating. The chemical composition of individual crystals was determined by energy dispersive spectrometry (EDS, 80 mm^2^ X-max detector, Oxford Instruments) at an accelerating voltage of 10 kV. Nitrogen adsorption-desorption data were recorded with an ASAP 2010 equipment from Micrometrics to determine the BET specific surface area, total pore volume and micropore volume of the raw materials and reaction products, as well as the reference crystals. The weight percentage of solid retentate after aging was determined by filtration through a 1 μm filter paper and gravimetric method, while the filtrates were analyzed by ICP-SFMS.

## Results

### Characterization of the starting materials

**Figure 1:**
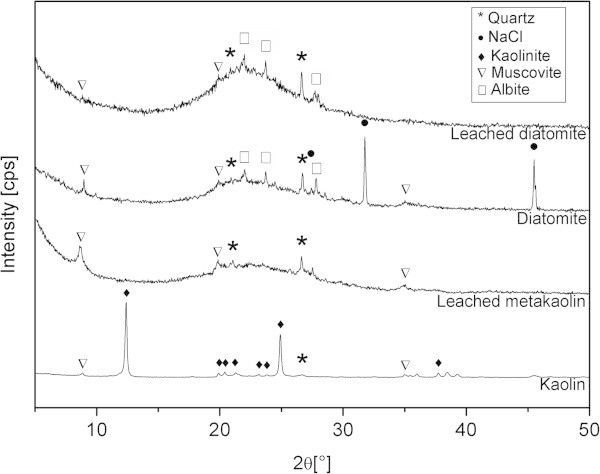
**XRD diffractograms of the raw materials and acid-leached materials.**

X-ray diffractograms of the raw aluminosilicates and dealuminated counterparts are shown in Figure 1. Kaolin of course contains mostly kaolinite (evidenced by reflections at 2*θ* = 12.33; 19.80; 20.40; 21.40. 24.81 and 35.11) but also traces of quartz (2*θ* = 20.85; 26.66) and muscovite (2*θ* = 8.83; 35.06). Kaolin after calcination and leaching was mostly an amorphous material with weak characteristic peaks of muscovite and quartz. On the other hand, raw diatomaceous earth shows the occurrence of halite NaCl (2*θ* = 27.41; 31.76; 45.53), muscovite (2*θ* = 8.83; 27.83; 35,06 ), albite (2*θ* = 22.03; 23.70) and quartz (2*θ* = 20.85; 26.66) in addition to amorphous material. After acid treatment and the subsequent washing, the amorphous material remained and NaCl was removed, but the other minor constituents were still present (muscovite, albite and quartz).

The chemical compositions of the raw and leached materials measured by ICP-SFMS are given in Table 1. Raw kaolin and diatomaceous earth had a SiO_2_/Al_2_O_3_ molar ratio of 2.2 and 15, respectively. This ratio was successfully increased by acid leaching to 33 and 44 for kaolin and diatomaceous earth, respectively. Acid leaching also reduced significantly the concentration of impurities in both materials. Finally, the leached materials had comparable compositions in terms of magnesium, potassium and iron. However, leached diatomaceous earth was approximately 4 times richer in sodium and calcium, which can be understood from the presence of NaCl and calcium compounds in the raw diatomaceous earth originating from a region close the salt lake Uyuni.

**Table 1:** **Compositions (in mole%) of kaolin, diatomaceous earth, leached metakaolin, leached diatomaceous earth and ZSM-5 products by ICP-SFMS**

Composition	Kaolin	Leached metakaolin	ZSM-5 (K)	Diatomaceous earth	Leached diatomaceous earth	ZSM-5 (D)
SiO_2_	67.7	95.9	94.0	78.8	96.4	96.0
Al_2_O_3_	30.1	2.92	4.15	5.22	2.17	2.40
CaO	0.15	0.12	0.10	4.44	0.49	0.63
Fe_2_O_3_	0.37	0.16	0.18	0.22	0.06	0.07
K_2_O	1.13	0.60	0.65	1.29	0.33	0.30
MgO	0.59	0.19	0.22	3.30	0.19	0.23
Na_2_O	0.16	0.08	0.65	6.78	0.35	0.37
**Mol SiO**_**2**_ **/Al**_**2**_ **O**_**3**_	**2.2**	**33**	**23**	**15**	**44**	**40**

**Figure 2:**
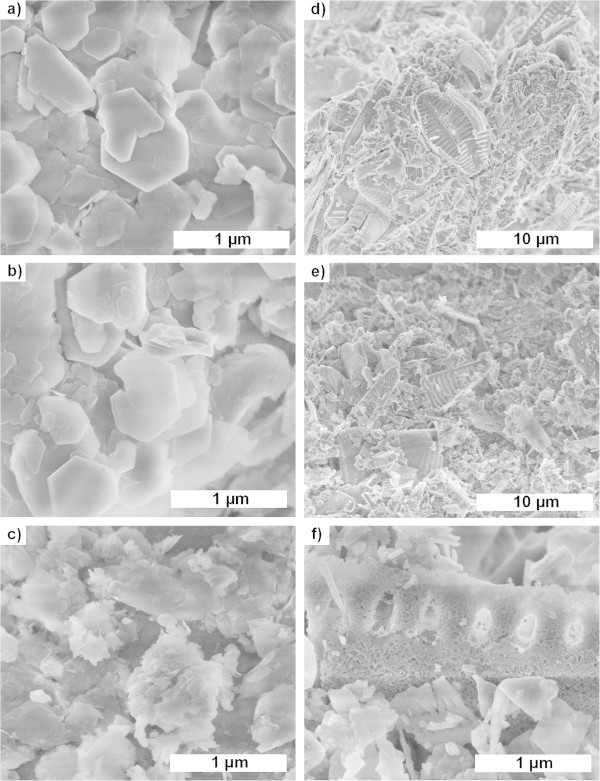
**Morphology of the raw materials and leached materials. (a)** Kaolin (50000x magnification). **(b)** Leached metakaolin (LMK) (50000x magnification). **(c)** Solid part of LMK after aging (50000x magnification). **(d)** Diatomaceous earth (5000x magnification). **(e)** Leached diatomaceous earth (LD) (50000x magnification). **(f)** Solid part of LD after aging (50000x magnification).

Figure 2 shows the morphology of the raw materials and leached materials revealed by SEM. Kaolin is composed of stacks of platelets with hexagonal symmetry which is typical of natural kaolinites (Figure 2(a)). The leached metakaolin (Figure 2(b)) has very similar platelet morphology but the surface area increased from 12 to 288 m^2^/g as presented in Table 2. This is not surprising since acid-leached metakolin is known to form microporous silica (Madhusoodana et al. 2001); (Zhang et al. 2007). Raw diatomaceous earth (Figure 2(d)) exhibited large particles with typical shapes of diatomaceous biogenic sediments. Some diatomaceous earth particles were partially broken in smaller pieces by the mechanical action of stirring during the acid treatment but their characteristic shapes could still be distinguished (Figure 2(e)). Leaching of diatomaceous earth only caused a slight increase in specific surface area (from 38 to 55 m^2^/g; Table 2).

**Table 2:** **Surface area and pore volumes derived from nitrogen adsorption data for the raw, leached materials, final products and standard sample**

Sample	BET surface area (m ^2^/g)	Total pore volume (cm ^3^/g)	Micropore volume (cm ^3^/g)
Kaolin	12	0.058	0.004
Leached metakaolin	288	0.24	0.089
Diatomaceous earth	38	0.093	0.003
Leached diatomaceous earth	55	0.11	0.006
ZSM-5 (K)	255 (82%)	0.17	0.082 (68%)
ZSM-5 (D)	298 (96%)	0.15	0.098 (82%)
ZSM-5 standard	310	0.15	0.12

### Hydrothermal synthesis

**Figure 3:**
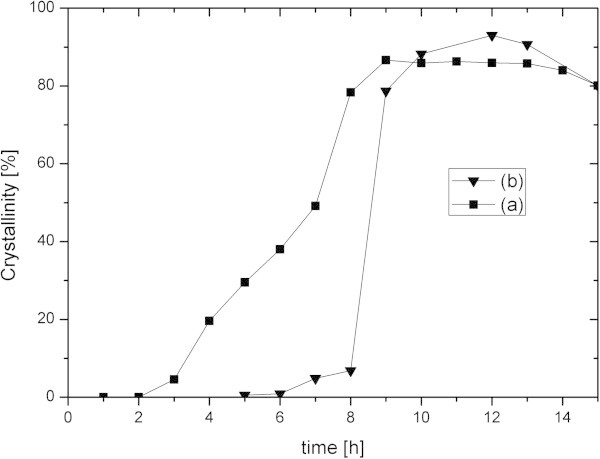
**Crystallinity as a function of time of the reaction products prepared from acid leached materials. (a)** Leached metakaolin. **(b)** Leached diatomaceous earth.

Hydrothermal synthesis in terms of composition of the synthesis mixture and synthesis time was first optimized to maximize the yield of ZSM-5 using leached metakaolin as a raw material. The composition used in this work was found to produce the highest yield of ZSM-5 crystals. Figure 3 shows the evolution of XRD crystallinity compared with a reference sample composed of 6–10 μm ZSM-5 crystals. It can be noticed in Figure 3(a) that samples prepared from leached metakaolin reached maximum crystallinity for synthesis times between 9 and 12 hours before decreasing for prolonged hydrothermal treatments. The reaction parameters of the dealumination and hydrothermal treatments obtained on kaolin were employed for diatomaceous earth. As shown in Figure 3(b), the best crystallinity for leached diatomaceous earth was obtained for 12 hours of synthesis.

### Characterization of the crystalline products

**Figure 4:**
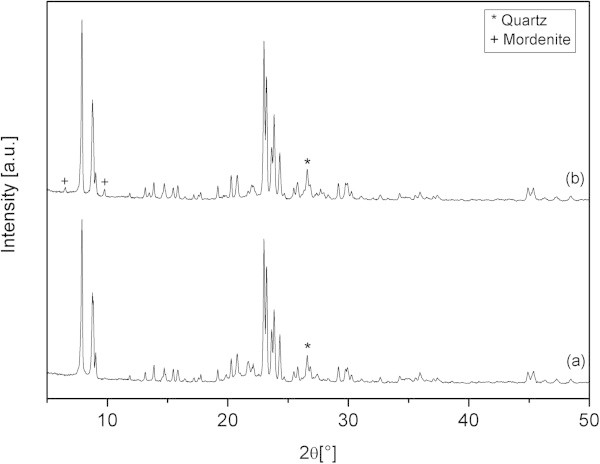
**XRD diffractograms of the products obtained after 12 hours of synthesis from acid leached materials. (a)** Leached metakaolin. **(b)** Leached diatomaceous earth.

The diffractograms of the final reaction products obtained from both types of raw materials after 12 h synthesis are presented in Figure 4. The main characteristic peaks correspond to the MFI structure (2*θ* = 7.9; 8.7; 23.0 etc.) in good agreement with the reference pattern PDF-042-0024. The intensities of the main peak of quartz are similar in both samples and of the same order of magnitude as in the leached materials. Therefore, the quartz content is similar in both samples and originates from the raw materials. However, the reaction product obtained from diatomaceous earth contained traces of mordenite, approximately 5% of the intensity of the main peak of ZSM-5. The composition of the final products after 12 h synthesis was determined by ICP-SFMS analysis and the results were presented in Table 1. The average SiO_2_/Al_2_O_3_ molar ratio was 23 and 40 for the reaction products obtained from leached metakaolin and diatomaceous earth, respectively. From these data, the reaction products could be considered as quite pure ZSM-5 with traces of mordenite formed during synthesis and of quartz remaining from the raw material.

**Figure 5:**
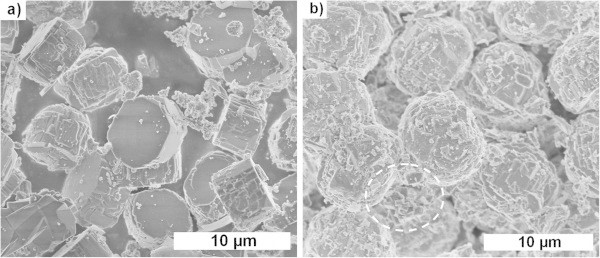
**SEM images of ZSM-5 crystals from kaolin and diatomaceous earth. (a)** Kaolin (5000x magnification). **(b)** Diatomaceous earth (5000x magnification).

The morphology of the reaction products was studied by SEM and typical images were presented in Figure 5. Synthesis from leached metakaolin resulted in the formation of flat tablet shaped ZSM-5 crystals with a diameter of 5–6 μm, but also some smaller particles, as shown in Figure 5(a). In contrast, the ZSM-5 crystals obtained from leached diatomaceous earth were rounded with average diameter around 7–8 μm and aspect ratio close to 1 (Figure 5(b)). This sample also contained smaller particles and particularly small slabs as those encircled in Figure 5(b), which were attributed to mordenite.

## Discussion

As shown above, the combination of both sodium hydroxide and *n*-butylamine together with leached metakaolin or leached diatomaceous earth was efficient to produce micron-sized ZSM-5 crystals within similar synthesis times. However, the reaction mechanism seems to differ depending on which alumino-silica source was used. Figure 3(a) clearly shows that crystal growth is triggered after an induction period of 2 h and slowly progresses until maximum crystallinity is reached after 9 h when leached metakaolin was used. In contrast, the induction period was extended to 6 h before a sudden burst of crystal growth occurred between 8 and 9 h, if leached diatomaceous earth was used as alumino-silica source (Figure 3(b)). The differences in growth rates are not yet understood. However, the difference in induction period between both synthesis mixtures may be related to the state of the synthesis mixtures after 24 h aging and before hydrothermal treatment. After filtration through a 1-μm filter paper, 26 and 80 wt.% of the original solid material remained from the aged synthesis mixtures prepared from leached metakaolin and leached diatomaceous earth, respectively. The filtrates were found to be silica-rich sols by ICP-SFMS (SiO_2_/Al_2_O_3_ molar ratio ~ 400–800). As shown in Figure 2(c), the solid retentate of the aged synthesis mixture prepared from leached metakaolin consisted of poorly defined platelets with a SiO_2_/Al_2_O_3_ molar ratio of 7.5, which probably stem from undigested muscovite or other materials that did not become microporous or possibly sintered upon calcination. On the other hand in Figure 2(f), particles with typical morphology of fossilized diatom still comprised the main constituent of the diatomaceous earth synthesis mixture after aging, even though further communition occurred by mechanical mixing during aging. Therefore, the longer induction time encountered for the leached diatomaceous earth system can be imparted to the heavily condensed state still present after aging in comparison to the silica-rich sol resulting from aging of the leached metakaolin mixture, the latter being more homogeneous and requiring less transformation for nucleation of zeolite crystals.

Although induction time was longer, the maximum crystallinity was slightly higher for samples prepared from diatomaceous earth than from kaolin and amounts to 93 and 87%, respectively, as shown in Figure 3. By a normalization of the BET specific surface area and total micropore volume with respect to the ZSM-5 standard sample also used for determining crystallinity by XRD, we show that the crystallinity of the reaction product obtained from kaolin is in good agreement with the values given in Table 2 with a specific surface area of 82%. The total micropore volume (68%) value indicates that the final product prepared from kaolin contains approximately 30% of non-microporous material in addition to the ZSM-5 crystals. The same values calculated from the BET specific surface area and total micropore volume for the diatomaceous earth-derived product, 96 and 82% respectively, are higher than that obtained by XRD (93%). This can be attributed to the presence of mordenite as a by-product in addition to non-microporous materials.

**Figure 6:**
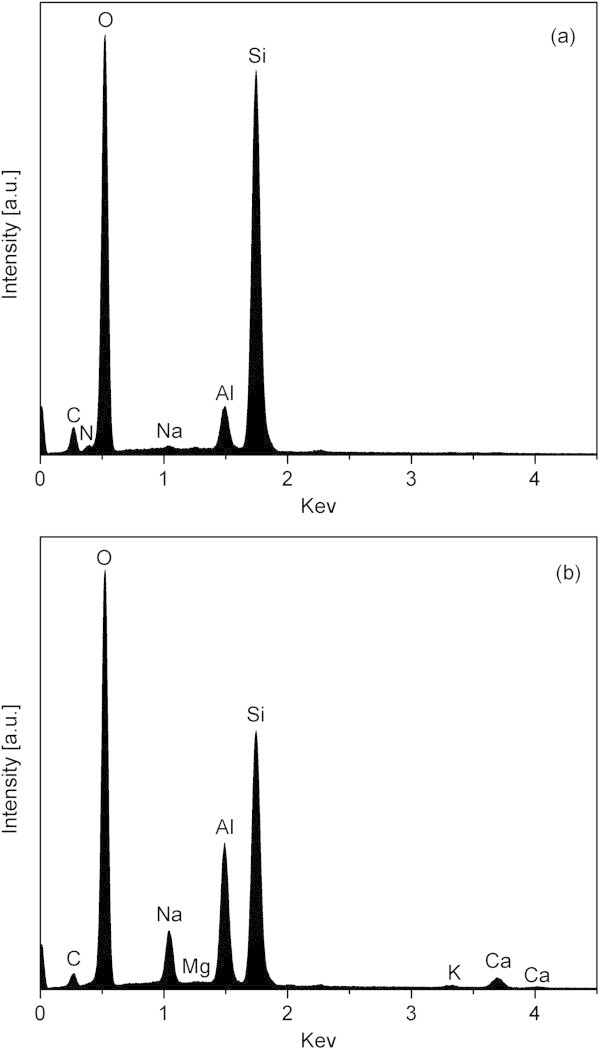
**EDS spectra of the final product obtained from leached diatomaceous earth. (a)** ZSM-5 crystal. **(b)** Mordenite crystal.

It was not possible to prevent the formation of mordenite by further optimization of the synthesis parameters. Instead, formation of mordenite occurred randomly, probably due to the variability of the diatomaceous earth raw material. Calcium was found to be concentrated in the mordenite crystals as revealed by the comparison of the EDS spectra between uncalcined ZSM-5 (Figure 6(a)) and mordenite (Figure 6(b)) crystals. Therefore, the higher calcium content in leached diatomaceous earth as compared to leached kaolin probably favored the formation of mordenite. The presence of *n*-butylamine as templating agent in the ZSM-5 crystals was also confirmed by EDS, as shown in Figure 6(a) with the characteristic peak of nitrogen and carbon, while that of sodium was quite weak.

The BET specific surface area obtained in this work for the sample prepared from leached diatomaceous earth (298 m^2^/g) is comparable with that obtained in the study by Sang et al. ^9^ (294 m^2^/g), who employed water glass and aluminum sulfate as Si and Al sources, respectively. Therefore, Bolivian diatomaceous earth appears as a competitive source of inexpensive raw materials for the synthesis of ZSM-5 crystals. In addition to the higher crystallinity and BET specific surface area achieved in this work compared with kaolin, diatomaceous earth does not require heat treatment at high temperature for metakaolinization.

## Conclusions

The inexpensive raw materials: kaolin, Bolivian diatomaceous earth, sodium hydroxide and *n-*butylamine have been used to prepare ZSM-5 zeolites with SiO_2_/Al_2_O_3_ molar ratios in the range 20 – 40. Dealumination of the raw materials by acid leaching made it possible to reach appropriate SiO_2_/Al_2_O_3_ molar ratios and to reduce the amount of iron and other impurities in the raw materials. After mixing and aging for 24 hours, synthesis by hydrothermal treatment was carried out at165°C either using leached metakaolin or leached diatomaceous earth as source of alumino-silica. The results clearly show for the first time that well-crystallized ZSM-5 can be directly prepared from both materials in combination with sodium hydroxide and *n*-butylamine under appropriate synthesis conditions. Reaction mixtures prepared from leached diatomaceous earth showed longer induction period due to the slower digestion of the fossilized diatom skeletons compared with microporous leached metakaolin. However, the use of leached diatomaceous earth allowed higher yield in ZSM-5 crystals within comparable synthesis times despite the formation of low contents of mordenite, which was related to the high calcium content of diatomaceous earth. Another considerable advantage of diatomaceous earth over kaolin is that diatomaceous earth does not require heat treatment at high temperature for metakaolinization.
